# Supplementary material for: Molecular mechanisms of drug resistance and compensation in SARS-CoV-2 main protease: the interplay between E166 and L50
Source: mBio. 2025 Apr 4;16(5):e04068-24. doi: 10.1128/mbio.04068-24 (PMC12077200; doi:10.1128/mbio.04068-24)
Supplement: Supplemental Material — Figures S1-S3 and Tables S1 and S2. [file mbio.04068-24-s0001.pdf]

SUPPLEMENTAL MATERIAL

Molecular Mechanisms of Drug Resistance and Compensation in SARS-CoV-2 Main Protease: The Interplay Between E166 and L50

Sarah N. Zvornicanin[1], Ala M. Shaqra[1], Julia Flynn[1], Heidi Carias Martinez[2], Weiping Jia[2], Stephanie Moquin[2], Dustin Dovala[2], Daniel N. Bolon[1], Nese Kurt Yilmaz[1] and Celia A. Schiffer[1,*]

[1] Department of Biochemistry and Molecular Biotechnology, University of Massachusetts Chan Medical School, Worcester, Massachusetts 01605, USA

[2] Biomedical Research, Novartis, Emeryville, CA 94608, USA

*Correspondence: Celia.Schiffer@umassmed.edu

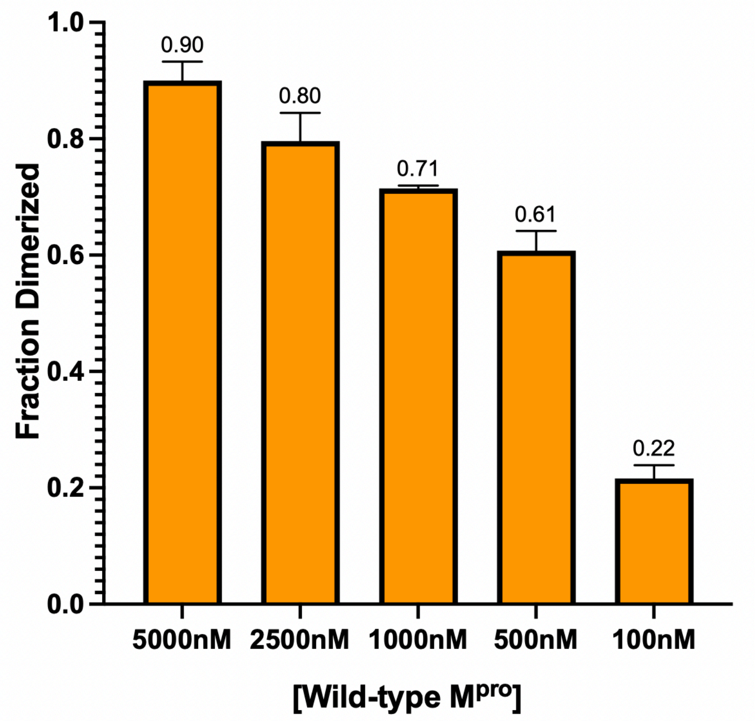

Figure S1: Fraction of dimeric protein as a function of concentration for WT M^{pro}, determined by native mass spectrometry.

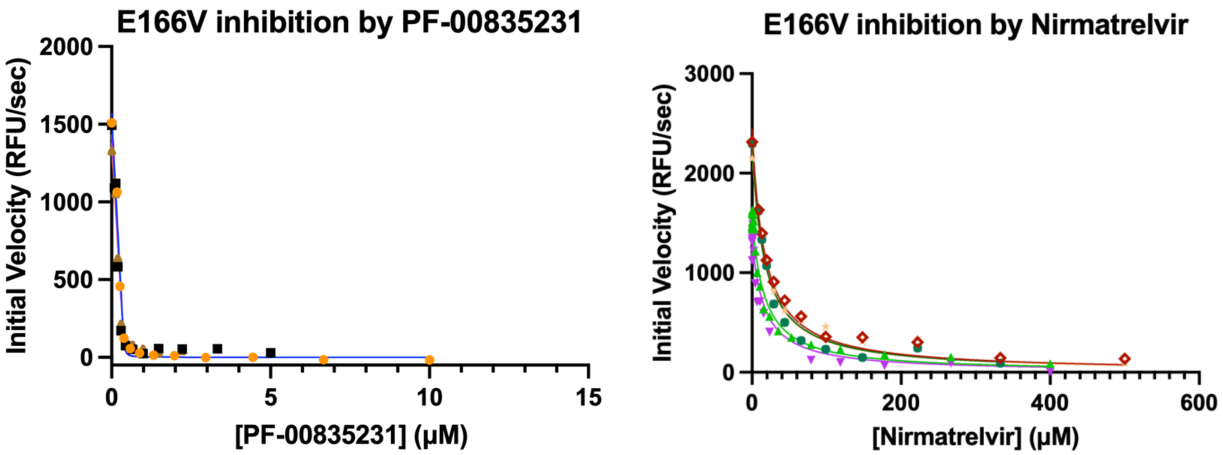

Figure S2: Inhibition curves for the resistant E166V M^{pro} variant to determine the inhibitor concentration to ensure saturation of the enzymes in the subsequent thermostability measurements (see Table S1).

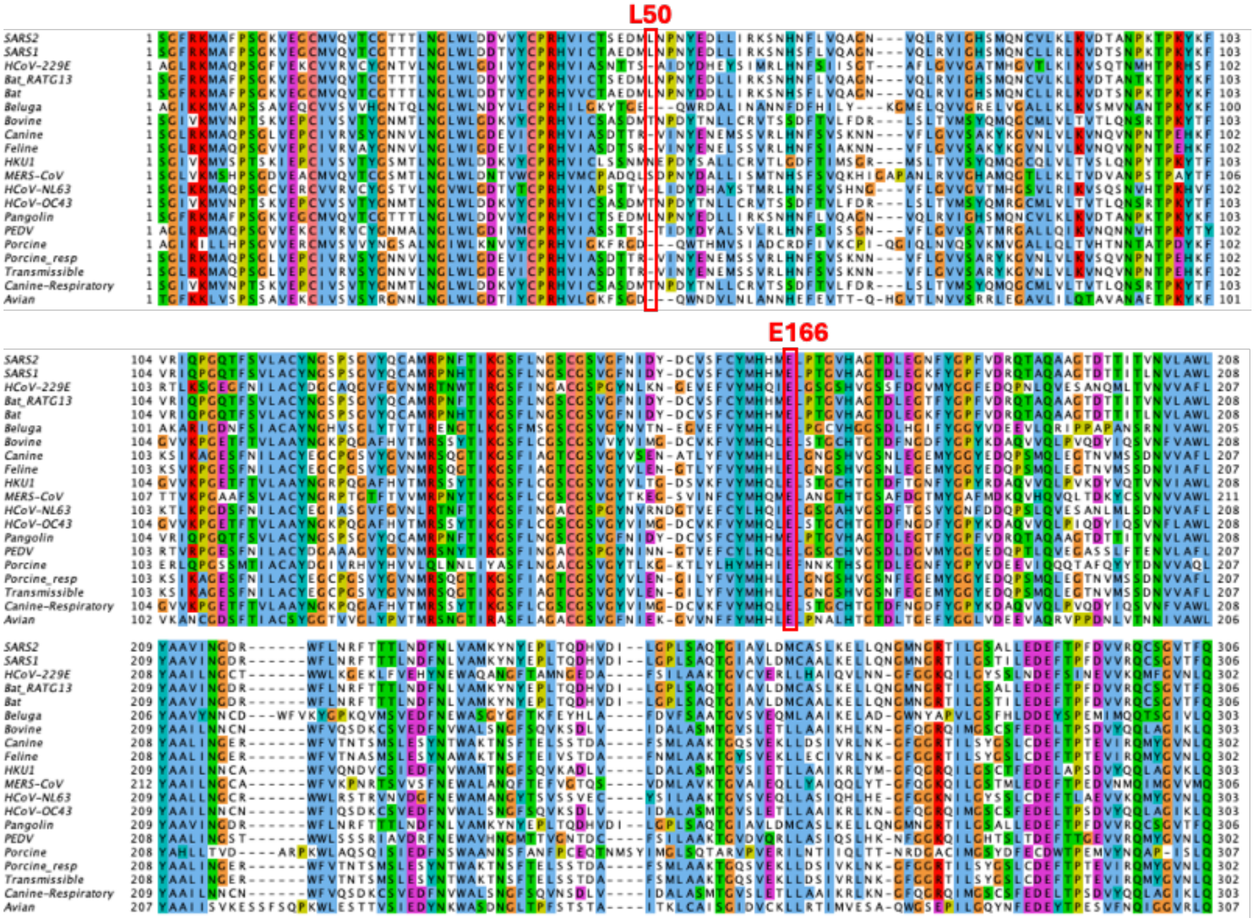

Figure S3: Multiple sequence alignment of M^{pro} from various coronaviruses, showing the evolutionary conservation of E166 and variability of the L50 position. Residues are colored by Clustal X default coloring in Jalview.

Table S1. Stability of M^{pro} variants assessed by melting temperature T_m (°C) in the absence (apo) and presence of inhibitors. The protein was at 2 μM, nirmatrelvir at 400 μM, and PF-00835231 at 20 μM to ensure complete saturation for all variants.

M^{pro} Variant	Apo T_m (°C) ± SEM	Nirmatrelvir T_m (°C) ± SEM	PF-00835231 T_m (°C) ± SEM
WT	51.12 ± 0.04	72.7 ± 0.2	66.9 ± 0.1
L50F	50.80 ± 0.01	73.5 ± 0.2	68.6 ± 0.3
E166A	49.6 ± 0.1	63.4 ± 0.2	57.7 ± 0.1
E166V	50.0 ± 0.1	53.3 ± 0.1	58.78 ± 0.07
E166A/L50F	49.1 ± 0.1	63.7 ± 0.1	58.59 ± 0.05
E166V/L50F	49.6 ± 0.1	53.7 ± 0.1	59.50 ± 0.03

Table S2. Crystallization and refinement statistics of SARS-CoV-2 Mpro variants E166A, E166V, and E166V/L50F in complex with PF-00835231.

SARS-CoV-2 M^{pro} Variant: Inhibitor	E166A: PF-00835231	E166V: PF-00835231	E166V/L50F: PF-00835231
PDB ID	9EL4	9ELV	9MEI
**DATA COLLECTION**			
Location	NLS-II, Synchrotron	NLS-II, Synchrotron	NLS-II, Synchrotron
Resolution Range (Å)	31.65 - 1.88 (1.947 - 1.88)	33.74 - 1.62 (1.678 - 1.62)	33.53 - 1.84 (1.906 - 1.84)
Space Group	P 1 21 1	P 1 21 1	P 1 21 1
a, b, c, (Å)	54.633, 99.272, 58.374	55.321, 99.001, 58.833	55.279, 99.464, 58.983
α, β, γ (°)	90, 107.438, 90	90, 108.04, 90	90, 108.021, 90
Total Reflections	94022 (9300)	152454 (15207)	102271 (10144)
Unique Reflections	47107 (4653)	76320 (7609)	51342 (5075)
Multiplicity	2.0 (2.0)	2.0 (2.0)	2.0 (2.0)
Completeness (%)	97.59 (96.53)	99.92 (99.80)	97.68 (96.57)
Average I/σ	17.53 (5.96)	17.16 (2.42)	13.60 (3.60)
Wilson B-Factor	24.69	21.79	24.76
R_{merge}^{a}	0.02568 (0.1097)	0.01942 (0.2783)	0.03109 (0.1955)
$CC_{1/2}$	0.999 (0.974)	1.000(0.867)	0.998(0.93)
**REFINEMENT**			
R_{factor}^{c}	0.1577 (0.1819)	0.1683 (0.2823)	0.1692 (0.2266)
R_{free}^{d}	0.2023 (0.2647)	0.2034 (0.3829)	0.2188 (0.2266)
**RMSD[b] in:**			
Bond Lengths (Å)	0.08	0.02	0.01
Bond Angles (°)	0.832	1.35	2.33
**Ramachandran:**			
Favored (%)	98.01	98.33	97.67
Allowed (%)	1.66	1.67	2.33
Outliers (%)	0.33	0.00	0.00
Rotamer outliers (%)	0.77	0.58	0.00
**B-Factors:**			
Average	30.62	29.52	31.19
Macromolecules	29.19	27.80	30.06
Ligand	27.50	30.18	33.20
Solvent	41.79	41.89	41.21

[a]$R_{sym} = \Sigma \mid I - \langle I \rangle \mid / \Sigma I$, where I = observed intensity,
$\langle I \rangle$ = average intensity over symmetry equivalent.

[b]RMSD, root mean square deviation.

[c]$R_{factor} = \Sigma \,||F_o| - |F_c||/ \Sigma|F_o|$.

[d]R_{free} was calculated from 5% of reflections, chosen randomly, which were omitted from the refinement process.

Statistics for the highest-resolution shell are shown in parentheses.
